# Impact of smoking on health-related quality of Life after percutaneous coronary intervention treated with drug-eluting stents: a longitudinal observational study

**DOI:** 10.1186/s12955-016-0578-4

**Published:** 2017-01-03

**Authors:** Chao Xue, Lin Bian, Yu Shui Xie, Zhao Fang Yin, Zuo Jun Xu, Qi Zhi Chen, Hui Li Zhang, Chang Qian Wang

**Affiliations:** Department of Cardiology, Shanghai Ninth People’s Hospital, Jiao Tong University School of Medicine, Shanghai, 200011 People’s Republic of China

**Keywords:** Smoking, Quality of life, Drug-eluting stent, Percutaneous coronary intervention

## Abstract

**Background:**

Smoking has been shown to reduce health-related quality of life (HRQOL) in patients with coronary artery disease (CAD) undergoing percutanous coronary intervention (PCI) either by means of balloon angioplasty or with the use of bare-metal stents (BMS). Drug-eluting stents (DES) have now been widely used and are related to substantial reduction of restenosis and significantly improved HRQOL compared with BMS. This study aimed to evaluate the effects of smoking on HRQOL in patients after PCI in DES era.

**Methods:**

A cohort of 649 patients admitted for CAD and treated with drug-eluting stents were included in this prospective, observational study. Patients were classified as non-smokers (*n* = 351, 54.1%), quitters (*n* = 126, 19,4%), or persistent smokers (*n* = 172, 26.5%) according to their smoking status at the time they first admitted to hospital and during the first year of follow-up. Each patient was prospectively interviewed at baseline, 6 months and 1 year following PCI. HRQOL was assessed with the use of Medical Outcomes Study 36-Item Short-Form Health Survey (SF-36).

**Results:**

For the overall population, HRQOL scores at 1-year follow-up were significantly higher than baseline for all 8 domains. At 1-year follow-up, the HRQOL scores in persistent smokers were still lower than that in non-smokers in 6 domains except for bodily pain and mental health, and than that in quitters in 5 domains except for bodily pain, role emotional and mental health. There were no significant differences with regard to the scores between non-smokers and quitters except role emotional for which non-smokers had higher scores. After adjustment, persistent smokers demonstrated significantly less improvements in HRQOL than non-smokers in 6 domains except for bodily pain and social functioning and significantly less improvement than quitters for general health. Improvements of quitters were comparable to that of non-smokers in all domains. Multivariate linear regression analyses showed persistent smoking was an independent risk factor for PCS and MCS improvements.

**Conclusions:**

Persistent smoking substantially diminishes the potential quality-of-life benefits of DES. Efforts should be made to promote smoking cessation after DES implantation which could greatly improve the health quality outcomes.

## Background

Coronary artery disease (CAD) has been the second leading cause of death in the world including China. This disease not only increases the mortality but also affects the health-related quality of life (HRQOL) severely, exerting negative effects on the energy and vitality levels, social interactions and psychological aspects. Percutaneous coronary interventions (PCI) had been shown to effectively reduce mortality and morbidity in patients with CAD [1]. The continued evolution of PCI techniques, especially the introduction of using drug-eluting stents (DES), has reduced the incidence of coronary restenosis and the need for target vessel revascularization [2, 3].

Although considerable studies have been directed at improving the outcomes of PCI, these studies have generally focused on “hard” end points such as death or nonfatal myocardial infarction. In fact, HRQOL has also played an very important role in the management of CAD patients, which has been shown to predict adverse clinical outcomes [4, 5]. Assessment of HRQOL and its determinants may help bridge the gap between research and clinical practice [6].

One of the most significant modifiable cardiovascular determinants which is linked with poorer outcomes after PCI is cigarette smoking. Previous studies have shown that long-term risks of myocardial infarction and death are higher in smokers than in nonsmokers after PCI [7]. However, the specific effects of cigarette smoking on overall HRQOL after PCI especially with DES have not been comprehensively studied. The purpose of this study was to compare the effect of smoking on HRQOL in patients with CAD treated with DES.

## Methods

### Study population

Consecutive patients with a discharge diagnosis of CAD for the first time (including stable angina, unstable angina and myocardial infarction with or without ST-segment elevation) who underwent PCI with DES in people’s hospital were enrolled between May 2011 and November 2013. All patients received optimal medical therapy. Patients were excluded if they refused to participate or if they were physically incapable of responding to a questionnaire. Patients died in hospital or accompany with other disease such as rheumatoid arthritis which limit the physical activity were also excluded.

### Evaluation of HRQOL

Researchers conducted chart reviews for each study subject at the time of enrollment and HRQOL was evaluated at baseline and at 6 and 12 months after revascularization in patients treated with DES. The baseline questionnaires were completed in hospital at the time of the initial revascularization procedure, subsequent questionnaires were sent by mail. Those patients who did not respond to the mailed survey more than 2 weeks were contacted by telephones. We relied on previously validated questionnaire namely the Short-Form 36 (SF-36) health survey [8] to assess the patients overall healthy perception. This general HRQOL instrument was chosen rather than more specific tools since it provides an assessment of subjects’ own perception of their quality of life as a function of their general state of health. SF-36 includes 36-item scales measuring the following 8 health domains: physical functioning, role limitations due to physical problems, bodily pain, general health perceptions, vitality, social function, role limitations due to emotional problems, and mental health, as well as health change over the past year. Summary scores are derived by collapsing the 8 subscales, each scale ranges from 0 to 100, with a higher score corresponding to a better HRQOL. The 8 specific domains of physical and emotional scores can be summarized into 2 main scores: the Physical Component Score (PCS) and Mental Component Score (MCS).

### Smoking status evaluation

Smoking status was assessed on the basis of information obtained from hospital medical records at the time of first medical examination and rechecked by telephone interview. All patients were classified as non-smokers, quitters, or persistent smokers. Smokers were defined as patients who had smoked cigarette for at least 1 year and still smoked or at least smoking within 1 month before baseline interview [9]. Non-smokers defined as patients who had never smoked cigarettes regularly [10]. Patients who smoked before baseline interview and continued smoking during the follow-up period were considered persistent smokers. Patients who smoked during the year before baseline interview but stopped smoking during the follow-up period were considered quitters. No patients in the study began smoking after the index revascularization procedure or relapsed to smoke after 1 year of abstinence.

### Clinical follow-up

All patients received clinical follow-up at 1, 6 and 12 months after discharge to determine their symptomatic and clinical status. All end points including death, recurrent myocardial infarction (MI), stroke or repeat revascularization were recorded through direct patient interview in a special outpatient clinic or by indirect conversation with patients. Relatives had to be contacted by phones for clinical events follow-up if patients died.

### Statistical analysis

Continuous variables were expressed as mean ± SD and differences among the three groups of patients were tested for significance with one-way analyses of variance. Categorical variables were presented as counts and frequencies and compared with Chi-square test or the Fisher’s exact test.

A propensity score of probability in persistent smoking was used to adjust for potential bias between these groups. This was accomplished by performing a multivariable logistic regression analysis using persistent smoking as the dependent variable and entering all demographics, physical examination findings, clinical presentation and medications that were likely to affect the probability of persistent smoking. Stepwise backward elimination was employed and the resultant independent predictors of persistent smoking were then used to calculate the probability of persistent smoking (propensity score). By introducing the propensity score into regression adjustment, the effect of persistent smoking was estimated by adjustment for the impact of background covariates. The bias in the background covariates between these three groups could be removed by adjustments made with the propensity score [11, 12].

Multivariate linear regression models were created to identify whether the mean change of quality of life of persistent smokers differed from that of either never smokers or quitters. Each regression model adjusted for demographic characteristics (age, sex, marital status), comorbid medical conditions (hypertension, diabetes, hypercholesterolemia, congestive heart failure, impaired renal function, history of myocardial infarction), other clinical factors (number of disease vessels, lesion type, total stent length, ejection fraction). The propensity score was forced into all the models as covariate to balance the potential bias. Multiple imputation strategy was employed to account for missing scores which could potentially produce selection bias from survey non-responders. The results of sensitivity analysis using imputed data were similar to analytic cohort and were not presented in this paper.

Statistical analyses were performed using SPSS 19.0 for Windows (SPSS Inc, Chicago, Illinois). All tests of significance were two-tailed and a *P* <0.05 was considered significant.

## Results

### Patient population

A total of 785 consecutive patients were enrolled and completed the baseline HRQOL instrument. 649 (82.7%) finally completed the 1-year quality-of-life assessment and were enrolled into our study for subsequent analysis. Of them, 172 (26.5%) patients were persistent smokers, 351 (54.1%) patients were non-smokers and 126 (19.4%) patients were quitters. Nineteen patients died before 1-year interview. There were no significant differences between non-respondents and respondents in terms of gender and most baseline characteristics except that non-respondents were more likely to be younger (60.04 years vs. 62.84 years, *P* = 0.005) and were less likely to be married (78.7% vs. 87.2%, *P* = 0.011).

### Baseline characteristics and angiographic features

Baseline characteristics and angiographic features of these groups by smoking status were shown in Table 1. Smokers were significantly younger and more male gender than nonsmokers, and had higher body mass index. Smokers also had more diabetes mellitus, hypertension and prior acute myocardial infarction compared with non-smokers. Left ventricular ejection fraction, laboratory test and medical treatment were similar between smokers and non-smokers. There were no significant differences in angiographic and PCI parameters between these three groups.

**Table 1:** Baseline characteristics and angiographic features according to smoking status

Characteristic	Persisitent smokers (*N* = 172)	Never smokers (*N* = 351)	Quitters (*N* = 126)	*P* Value			
				3 Groups	Persistent vs Never	Persistent vs Quitter	Never vs Quitter
Age (years)	60.27 ± 9.65	65.05 ± 9.32	60.21 ± 9.12	<0.001	<0.001	0.956	<0.001
Male *n* (%)	155 (90.1)	211 (60.1)	118 (93.7)	<0.001	<0.001	0.277	<0.001
Marital status n (%)	147 (85.5)	310 (88.3)	114 (90.5)	0.405	0.356	0.195	0.509
Body mass index (kg/m^2^)	25.79 ± 2.80	25.12 ± 2.91	25.91 ± 2.74	0.005	0.011	0.739	0.008
Risk Factors n (%)							
Diabetes mellitus	33 (16.9)	89 (25.4)	19 (15.1)	0.015	0.029	0.679	0.021
Hypertension	105 (61.0)	244 (69.5)	81 (64.3)	0.003	0.002	0.718	0.019
Hypercholesterolemia	55 (32)	126 (35.9)	44 (34.9)	0.674	0.376	0.594	0.844
Heart failure	16 (9.3)	25 (7.12)	11 (8.73)	0.808	0.529	0.865	0.712
Impaired renal dysfunction	6 (3.5)	15 (4.3)	4 (3.2)	0.858	0.667	0.882	0.588
Prior AMI n (%)	30 (17.4)	36 (10.3)	23 (18.3)	0.021	0.020	0.856	0.019
Unstable angina	117 (68.0)	221 (63.0)	82 (65.1)	0.521	0.225	0.594	0.672
Left ventricular ejection fraction	65.52 ± 6.93	65.38 ± 8.84	66.28 ± 8.43	0.577	0.86	0.435	0.299
In-hospital laboratory test							
Fasting plasma glucose (mmol/L)	5.48 ± 1.55	5.77 ± 1.34	5.51 ± 1.26	0.170	0.125	0.477	0.229
Total cholesterol (mmol/L)	4.07 ± 0.88	4.21 ± 1.35	4.16 ± 0.87	0.422	0.190	0.522	0.651
Triglycerides (mmol/L)	1.67 ± 0.93	1.58 ± 1.15	1.75 ± 1.13	0.308	0.393	0.532	0.141
HDL cholesterol (mmol/L)	0.96 ± 0.19	0.99 ± 0.20	0.98 ± 0.14	0.136	0.046	0.319	0.505
LDL cholesterol (mmol/L)	2.43 ± 0.77	2.31 ± 0.82	2.44 ± 0.78	0.122	0.085	0.974	0.114
In-hospital treatments n (%)							
Antiplatalet	172 (100)	351 (100)	126 (100)	1.000	1.000	1.000	1.000
Statins	169 (98.3)	348 (99.1)	123 (97.6)	0.441	0.370	0.699	0.187
ACEI/ARB	110 (64.0)	215 (61.3)	82 (65.1)	0.693	0.550	0.841	0.447
CCB	31 (18.0)	74 (21.1)	26 (20.6)	0.708	0.412	0.571	0.916
β-blockers	87 (50.6)	190 (54.1)	66 (52.4)	0.742	0.445	0.759	0.735
No. of diseased vessels n (%)				0.429	0.185	0.892	0.528
1	85 (49.4)	158 (45.0)	61 (48.4)				
2	50 (29.1)	91 (25.9)	35 (27.8)				
3	37 (21.5)	102 (29.1)	30 (23.8)				
Reference vessel diameter (mm)	2.95 ± 0.42	3.01 ± 0.40	2.96 ± 0.34	0.213	0.100	0.732	0.277
Percentage diameter stenosis (%)	85.82 ± 12.31	84.61 ± 11.22	86.86 ± 11.55	0.146	0.260	0.446	0.062
B2/C class lesion n, (%)	129 (75.0)	253 (72.1)	99 (78.6)	0.344	0.480	0.472	0.155
Predilation n,(%)	148 (86.0)	299 (85.2)	111 (88.1)	0.722	0.793	0.604	0.420
Stent diameter (mm)	3.02 ± 0.41	3.08 ± 0.39	3.02 ± 0.34	0.104	0.074	0.982	0.104
Total stent length (mm)	32.18 ± 6.53	32.36 ± 7.00	33.56 ± 6.57	0.431	0.196	0.439	0.775
Stent delivery pressure (atm)	14.50 ± 2.31	14.39 ± 2.26	14.68 ± 2.05	0.435	0.583	0.487	0.202
Postdilation n,(%)	57 (33.1)	106 (30.2)	45 (35.7)	0.491	0.495	0.644	0.254

*AMI* acute myocardial infarction, *HDL* high density lipid, *LDL* Low density lipid, *ACEI* angiotensin converting enzyme inhibitors, *ARB* angiotensin receptor blockers, *CCB* calcium channel blockers

### Clinical follow-up

At 1-year follow-up, there were no significant differences in the rates of recurrent myocardial infarction (1.7% for persistent smokers, 1.1% for never smokers and 1.6% for quitters, *P* = 0.734), target lesion revascularization (4.7% for persistent smokers, 3.1% for never smokers and 3.2% for quitters, *P* = 0.657), stroke (1.2% for persistent smokers, 0.3% for never smokers and 0.8% for quitters, *P* = 0.404) and any event (7.6% for persistent smokers, 4.6% for never smokers and 5.6% for quitters, *P* = 0.39) between these three groups during the initial hospitalization or the 1-year follow-up (data not shown in Tables).

### Health-related quality of life

For the overall population, HRQOL scores at 6-month follow-up were significantly higher than baseline for all 8 domains, with improvements ranging from 4.0 points for mental health to 22.9 for role physical. At 1-year follow-up, there were significantly further improvements of 6 domains ranging from 1.3 points for mental health to 4.8 points for role emotion than 6 months. The dimensions of health for which scores did not improve significantly were bodily pain and vitality (data not shown in Tables).

**Fig. 1:**
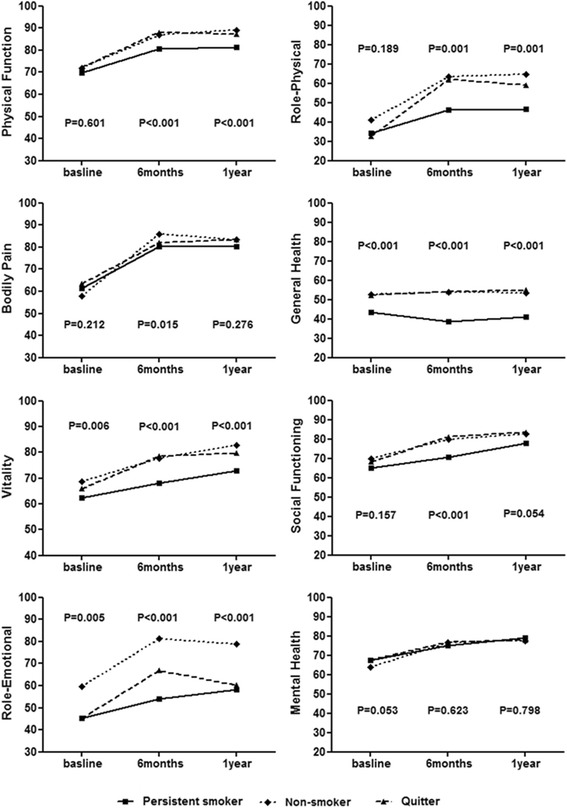
Unadjusted quality of life scores in 8 domains derived from SF-36 questionnaire according to smoking status

Table 2 and Fig. 1 showed the unadjusted life quality scores at baseline, 6 months and 1 year by smoking status. At baseline, the HRQOL was comparable between these three groups except for general health, vitality and role emotion, for which the non-smokers had higher HRQOL than the other two groups. There were no significant differences between the three groups in PCS and MCS.

**Table 2:** Unadjusted scores of health-related quality of life

SF-36 Subscale	Persisitent smokers (*N* = 172)	Never smokers (*N* = 351)	Quitters (*N* = 126)	*P* Value			
				3 Groups	Persistent vs Never	Persistent vs Quitter	Never vs Quitter
Baseline							
Physical functioning	69.87 ± 21.68	72.04 ± 23.36	72.24 ± 21.01	0.601	0.379	0.391	0.941
Role physical	34.33 ± 43.61	40.94 ± 41.15	32.65 ± 40.80	0.189	0.157	0.747	0.101
Bodily pain	61.25 ± 26.11	57.95 ± 27.93	63.38 ± 24.31	0.212	0.261	0.517	0.088
General health	43.33 ± 15.29	52.72 ± 16.50	52.28 ± 15.87	<0.001	<0.001	<0.001	0.820
PCS	52.20 ± 21.10	55.91 ± 21.65	55.14 ± 17.56	0.242	0.103	0.248	0.753
Vitality	62.26 ± 18.23	68.78 ± 19.17	65.91 ± 17.36	0.006	0.002	0.111	0.194
Social functioning	65.18 ± 22.67	70.02 ± 23.94	68.31 ± 20.70	0.157	0.056	0.268	0.531
Role emotional	45.33 ± 44.39	59.73 ± 45.99	45.32 ± 44.37	0.005	0.004	0.998	0.008
Mental health	67.6 ± 16.59	63.89 ± 15.99	67.61 ± 14.10	0.053	0.034	0.994	0.049
MCS	60.09 ± 20.73	65.60 ± 21.52	61.79 ± 19.43	0.050	0.017	0.511	0.126
6 months-baseline							
Physical functioning	80.55 ± 15.81	86.92 ± 19.30	88.13 ± 16.46	<0.001	0.001	<0.001	0.559
Role physical	46.34 ± 39.78	63.43 ± 44.65	62.00 ± 42.07	0.001	<0.001	0.003	0.780
Bodily pain	80.12 ± 16.70	85.98 ± 19.51	82.02 ± 19.11	0.015	0.005	0.410	0.076
General health	38.74 ± 18.36	53.88 ± 19.23	54.13 ± 17.34	<0.001	<0.001	<0.001	0.910
PCS	61.44 ± 17.71	72.55 ± 20.36	71.57 ± 18.68	<0.001	<0.001	<0.001	0.669
Vitality	67.97 ± 18.25	77.72 ± 19.28	78.51 ± 18.46	<0.001	<0.001	<0.001	0.727
Social functioning	70.57 ± 21.28	79.79 ± 21.07	81.22 ± 0.98	<0.001	<0.001	<0.001	0.573
Role emotional	54.11 ± 38.37	81.30 ± 45.63	66.91 ± 44.09	<0.001	<0.001	0.017	0.005
Mental health	75.11 ± 16.08	76.55 ± 15.82	76.82 ± 15.97	0.623	0.417	0.389	0.888
MCS	66.69 ± 19.63	78.84 ± 20.88	75.62 ± 21.03	<0.001	<0.001	0.001	0.192
1 year-baseline							
Physical functioning	81.43 + 16.95	89.19 ± 19.18	87.11 ± 16.32	<0.001	<0.001	0.010	0.328
Role physical	46.68 ± 39.75	64.67 ± 44.18	59.05 ± 41.39	0.001	<0.001	0.018	0.267
Bodily pain	80.37 ± 18.22	83.26 ± 17.36	83.32 ± 18.73	0.276	0.150	0.189	0.978
General health	41.13 ± 18.88	53.53 ± 19.06	54.87 ± 18.81	<0.001	<0.001	<0.001	0.555
PCS	62.40 ± 19.05	72.66 ± 20.31	71.09 ± 19.12	<0.001	<0.001	<0.001	0.054
Vitality	72.77 ± 20.83	82.72 ± 19.80	79.74 ± 19.63	<0.001	<0.001	0.006	0.218
Social functioning	77.73 ± 22.58	82.67 ± 21.02	83.49 ± 21.61	0.054	0.042	0.034	0.753
Role emotional	58.11 ± 37.48	78.72 ± 45.77	60.27 ± 39.42	<0.001	<0.001	0.675	<0.001
Mental health	78.89 ± 19.25	77.64 ± 16.65	77.84 ± 16.72	0.798	0.521	0.631	0.923
MCS	71.88 ± 21.67	80.44 ± 20.95	75.33 ± 19.51	0.001	<0.001	0.183	0.042

*PCS* physical component summary, *MCS* Mental component summary

Compared to non-smokers and quitters, persistent smokers had lower HRQOL scores at 6 months in 7 domains except for mental health and in 6 domains except for bodily pain and mental health separately. After risk adjustment, persistent smokers improved to a lesser extent than non-smokers in all the domains and than quitters in 4 domains except for bodily pain, social functioning, role emotion and mental health. Quitters and nonsmokers had comparable improvements on all SF-36 scales (Table 2).

At 1-year follow-up, the HRQOL scores in persistent smokers were still lower than that in non-smokers in 6 domains except for bodily pain and mental health, and than that in quitters in 5 domains except for bodily pain, role emotional and mental health. There were no significant differences with regard to the scores between non-smokers and quitters except role emotional for which non-smokers had higher scores. After adjustment, persistent smokers demonstrated significantly less improvement in HRQOL than non-smokers in 6 domains except for bodily pain and social functioning and significantly less improvement than quitters for general health. Improvements of quitters were comparable to that of non-smokers in all domains. Improvement in PCS and MCS also differed significantly according to smoking status (Table 3).

**Table 3:** Adjusted changes in Health-Related Quality of Life according to smoking status

SF-36 Subscale	Persisitent smokers (*N* = 172)	Never smokers (*N* = 351)	Quitters (*N* = 126)	*P* Value			
				3 Groups	Persistent vs Never	Persistent vs Quitter	Never vs Quitter
6 months-baseline							
Physical functioning	10.72 ± 15.62	15.62 ± 17.71	16.29 ± 16.74	0.003	0.002	0.005	0.703
Role physical	13.35 ± 50.05	24.66 ± 47.76	26.44 ± 43.18	0.020	0.011	0.019	0.719
Bodily pain	18.78 ± 24.92	24.19 ± 28.63	19.15 ± 27.60	0.053	0.035	0.910	0.078
General health	−4.86 ± 17.58	0.70 ± 14.57	2.49 ± 15.17	<0.001	<0.001	<0.001	0.268
PCS	9.50 ± 20.48	16.29 ± 19.91	16.09 ± 18.03	0.001	<0.001	0.004	0.922
Vitality	5.33 ± 16.09	9.68 ± 16.06	12.24 ± 14.92	0.001	0.003	<0.001	0.121
Social functioning	5.81 ± 21.19	10.92 ± 20.64	12.25 ± 24.70	0.015	0.011	0.554	0.554
Role emotional	8.31 ± 52.53	20.29 ± 50.33	19.72 ± 48.02	0.031	0.011	0.054	0.914
Mental health	7.28 ± 12.06	11.72 ± 15.34	9.72 ± 13.34	0.003	0.001	0.142	0.175
MCS	6.43 ± 20.00	13.15 ± 19.41	13.23 ± 17.94	<0.001	<0.001	0.003	0.968
1 year-baseline							
Physical functioning	11.87 ± 15.87	17.46 ± 18.25	15.84 ± 17.37	0.003	0.001	0.053	0.183
Role physical	14.99 ± 50.07	27.38 ± 42.64	24.35 ± 43.83	0.013	0.003	0.076	0.517
Bodily pain	19.60 ± 27.07	22.54 ± 26.55	20.23 ± 25.66	0.433	0.235	0.840	0.402
General health	−2.34 ± 17.09	0.81 ± 15.94	2.97 ± 16.26	0.017	0.039	0.006	0.203
PCS	11.03 ± 20.51	17.05 ± 18.20	15.85 ± 18.38	0.003	0.001	0.030	0.539
Vitality	10.14 ± 16.91	14.72 ± 16.51	13.31 ± 16.97	0.013	0.003	0.106	0.414
Social functioning	12.87 ± 21.62	14.30 ± 20.03	14.29 ± 25.53	0.760	0.477	0.577	0.994
Role emotional	11.86 ± 49.76	22.84 ± 50.93	12.98 ± 43.56	0.026	0.017	0.846	0.054
Mental health	10.79 ± 14.19	13.59 ± 16.15	10.49 ± 14.32	0.053	0.050	0.868	0.052
MCS	11.41 ± 20.88	16.36 ± 18.66	12.76 ± 17.77	0.013	0.006	0.546	0.070

*PCS* physical component summary, *MCS* Mental component summary

Multivariate linear regression analyses showed that age, persistent smoking, diabetic mellitus were the independent risk factors for PCS improvement and age, persistent smoker, marital status and diabetic mellitus were the independent risk factors for MCS improvement (Table 4).

**Table 4:** Regression analysis for HRQOL Changes at 1 year

Variables	PCS				MCS			
	β	95% CI		*P* value	β	95% CI		*P* value
		lower	upper			lower	upper	
Age	−0.262	−0.425	−0.099	0.002	−0.394	−0.555	−0.232	<0.001
Female	1.334	−2.497	5.184	0.492	1.061	−2.724	4.846	0.582
Marital status	1.141	−1.232	3.513	0.345	2.669	0.357	4.981	0.024
Persistent smoking	−6.521	−12.039	−1.004	0.021	−9.378	−14.886	−3.871	0.001
Hypertension	−2.616	−7.968	2.736	0.337	−1.872	−7.147	3.403	0.486
Diabetes Mellitus	−5.689	−10.922	−0.456	0.033	−7.120	−12.274	−1.966	0.007
Hypercholesterolemia	2.795	−2.912	8.501	0.101	1.767	−3.917	7.451	0.542
Heart failure	−0.281	−8.218	7.655	0.944	3.620	−4.201	11.442	0.364
Impaired renal dysfunction	3.875	−6.333	14.083	0.456	1.570	−8.415	11.554	0.758
Prior AMI	−1.884	−5.649	1.880	0.326	2.797	−0.913	6.508	0.139
Multivessel disease	−0.284	−6.567	5.999	0.929	−0.750	−6.942	5.443	0.812
Propensity Score	−0.043	−0.098	0.011	0.120	−0.045	−0.099	0.009	0.103

*HRQOL* health-related quality of life, *PCS* physical component summary, *MCS* mental component summary, *AMI* acute myocardial infarction

## Discussion

This study reveals that PCI is associated with significant improvements in most dimensions of health-related quality of life at 1 year for the overall population in DES era. However, even with DES implantation, persistent smoker had substantially less improvements in health status than non-smokers. Compared with persistent smokers, those patients who quit smoking after DES implantation had significantly greater improvements in quality of life. Multivariate analysis showed that persistent smoking was an independent risk factor for life quality improvement.

Previous studies have evaluated the relationship between health status and PCI in patients with coronary artery disease. Most studies, in which PCI was performed with the use of either balloon angioplasty or BMS, showed significant improvements of quality of life after the initial revascularization [13–16]. The revolutionary use of DES has further remarkably reduced the occurrence of restenosis thus the need of target lesion revascularization during follow-up. Recent studies revealed that PCI with DES could also improve the quality of life. Cohen et al. compared the outcomes of CABG with those of PCI with the use of DES among patients with three-vessel or left main coronary artery disease, and showed that both DES and CABG led to significant improvements in disease-specific and general health status over the course of 12 months [17]. van Domburg et al. evaluated the HRQOL following SES implantation in patients with multivessel disease and compared the outcomes with historical surgical and BMS arms of the ARTS-I study [18]. They found that along with a substantial reduction of restenosis, HRQOL after SES was significantly improved as compared with BMS. Our study, which was similar with previous studies, showed HRQOL scores at 1-year follow-up were significantly higher than baseline for the whole population after DES implantation.

The impact of smoking on HRQOL in the general population has been assessed by multiple cross-sectional and retrospective studies which have found that smokers tend to have worse quality of life than non-smokers [19, 20]. Several studies examined the relationship between smoking status and HRQOL derived from a medical intervention. Taira et al. evaluated the health status both at the time of index procedure and the first year of follow-up among 1432 patients including smokers, quitters and nonsmokers who received PTCA, and demonstrated that continued cigarette smoking has an adverse impact on the improvement in quality of life [21]. Recent study with a cohort of 2765 PCI patients by Jang et al. showed that smokers at the time of PCI have worse health status at 1 year than those who never smoked [22]. Our study, focusing on the health status in the DES era, showed that quality-of-life benefits brought by DES implantation are diminished by persistent smoking. Multivariate analyses showed that persistent smoking was an independent risk factor for PCS and MCS improvements.

The benefits of smoking cessation in patients with cardiovascular disease, including those undergoing PCI have been clearly established. The risks of adverse cardiac events would diminish in patients with CAD with smoking cessation and these benefits continued to increase over time after quitting [23]. Critchley et al. reviewed 20 studies with 12,603 smoking patients with ischemic heart disease in a meta-analysis and documented that cessation of smoking after acute myocardial infarction or cardiac surgery could significantly reduce mortality [24]. Buchanan et al. evaluated the association of smoking status with angina and HRQOL after acute myocardial infarction (AMI) and found that smokers who quitted after AMI had similar angina levels and mental health as non-smokers [25]. In our study, about 42.3% of smokers quitted after DES implantation, who have demonstrated significantly better improvement in HRQOL than persistent smokers and comparable improvement with nonsmokers. These findings were consistent with studies by Taira et al. and Jane et al. either treated with PTCA or by PCI, in which smoking cessation was documented to be associated with better health status outcomes [21, 22]. Considering the relatively low proportion of smoking cessation, efforts to stop smoking at the time of PCI should be made to improve the health outcomes of these procedures. These data including ours might supply further motivation for clinicians to stop smoking in patients treated with DES.

### Limitations

Our study has several limitations. The study is a single-center trial and the size of the study is small, the generalizability of our results is limited. Not all of the eligible population completed follow-up quality-of-life data. Only 82.7% of patients completed 1-year follow-up assessment. This respondent rate compares favorably with previous studies of quality-of-life outcomes. Non-respondents in our studies had similar scores of HRQOL with respondents at baseline. Treatment and adverse event rates were also similar between respondents and non-respondents. While interviewing patients’ relatives by phones for clinical follow-up may impact on the validity of the responses. Although adjustment for selection bias using propensity score, other factors such as socioeconomic factors and psychiatric conditions which may affect decision making and multiple dimensions of both physical and emotional health were not accounted for in this study. Further larger multicenter studies with long term follow-up should be needed to confirm these findings.

## Conclusions

PCI with DES implantation substantially improved HRQOL in patients with coronary artery disease, whereas these potential benefits could be diminished by persistent smoking. Patients who quit smoking after DES implantation demonstrated significantly better health status compared with persistent smokers. Efforts should be made to stop smoking at the time of PCI in order to improve the health outcomes.
